# Attentional Fluctuations, Cognitive Flexibility, and Bilingualism in Kindergarteners

**DOI:** 10.3390/bs9050058

**Published:** 2019-05-24

**Authors:** Stephanie L. Haft, Olga Kepinska, Jocelyn N. Caballero, Manuel Carreiras, Fumiko Hoeft

**Affiliations:** 1 Department of Psychiatry and Weill Institute for Neurosciences, University of California San Francisco, 401 Parnassus Ave., San Francisco, CA 94143, USA; stephanie.haft@berkeley.edu (S.L.H.); olga.kepinska@ucsf.edu (O.K.); jocelyn.caballero@ucsf.edu (J.N.C.); 2 Department of Psychology, University of California Berkeley, 2121 Berkeley Way, Berkeley, CA 94704, USA; 3 Basque Center on Cognition, Brain, and Language (BCBL), Mikeletegi Pasealekua 69, 20009 Donostia, Spain; m.carreiras@bcbl.eu; 4 IKERBASQUE, Basque Foundation for Science, Maria Diaz de Haro 3, 6 Solairua, 48013 Bilbao, Spain; 5 Departamento de Lengua Vasca y Comunicación, University of the Basque Country (UPV/EHU), Barrio Sarriena, 48940 Leioa, Spain; 6 Brain Imaging Research Center (BIRC), Institute for Brain and Cognitive Sciences (IBaCS), Departments of Psychological Sciences, Neuroscience and Psychiatry, University of Connecticut, 850 Bolton Road, Storrs, CT 06269, USA; 7 Haskins Laboratories, 300 George St #900, New Haven, CT 06511, USA; 8 Department of Neuropsychiatry, Keio University School of Medicine, 35 Shinanomachi Shinjuku, Tokyo 160-8582, Japan

**Keywords:** bilingualism, early childhood, attention, cognitive flexibility

## Abstract

The idea of a bilingual advantage in aspects of cognitive control—including cognitive flexibility, inhibition, working memory, and attention—is disputed. Using a sample of kindergarten children, the present study investigated associations between bilingualism and cognitive flexibility—a relationship that has shown mixed findings in prior literature. We also extend prior work by exploring relationships between bilingualism and attentional fluctuations, which represent consistency in attentional control and contribute to cognitive performance. To our knowledge, no previous study has explored this association. Theoretically, attentional fluctuations might mediate or moderate the relationship between bilingualism and cognitive flexibility. However, given evidence of null findings from extant literature when confounding variables are adequately controlled and tasks are standardized, we did not expect to find a bilingual advantage in either cognitive flexibility or attentional fluctuations. Our results supported this hypothesis when considering bilingualism both continuously and categorically. The importance of expanding upon mechanistic accounts connecting bilingualism to cognitive improvements is discussed.

## 1. Introduction

An explosion of empirical studies, reviews, and meta-analyses have addressed the proposed bilingual advantage in cognitive control (see [1,2,3,4,5,6,7,8,9,10]). In short, the bilingual advantage remains contentious, with research producing mixed and contradictory results. In making sense of these discrepant findings, there is general consensus that research should move away from confirming whether or not a bilingual advantage exists, and toward investigations of a priori hypotheses pertaining to specific circumstances or measures. The present study is a step in this direction, extending prior work by investigating if there is a relationship between bilingualism and a specific aspect of cognition: attentional fluctuations. Attentional fluctuations represent consistency in attentional control, predicting cognitive task performance in adults and in clinical populations [11,12]. However, the role of attentional fluctuations in children, and especially in bilingual children, is less clear. Accordingly, we examine bilingualism and attentional fluctuations in kindergarteners with a variety of language experiences, and we investigate bilingualism both continuously and dichotomously. We also examine bilingualism and cognitive flexibility given mixed findings on this relationship. To our knowledge, this is the first study to investigate whether bilingualism in young children relates to attentional fluctuations, which is an important aspect of cognitive control. 

### 1.1. The Controversy of the Bilingual Advantage in Cognitive Control

Interest in the cognitive consequences of bilingualism has soared in recent decades. Research has centered around the contention of a “bilingual advantage” in cognitive control, believed to result from the cognitive demands of managing two languages [13]. Cognitive control is a broad term referring to mechanisms controlling lower-level sensory, memory, or motor processes to achieve a common goal [14]. Typically, cognitive control is studied under the rubric of executive functions (EFs), which are commonly organized into components of cognitive flexibility, inhibitory control, and updating/monitoring [15].

There has been much excitement and support surrounding the bilingual advantage [16], which some researchers have suggested can even delay the onset of dementia [17]. However, other researchers have suggested that these results are inconsistent or even spurious [10,18], leading to an ongoing debate in the field. The full bilingual advantage controversy is too extensive to summarize exhaustively here—however, it is the main topic of several recent experimental studies [9,19], reviews [1,5,7,8], and meta-analyses [2,3,4,6,10]. 

Briefly, research supporting the bilingual advantage identifies inhibition and monitoring in bilinguals as potential mechanisms conferring enhanced cognitive control. This viewpoint holds that bilinguals have strong inhibition because they constantly have to suppress the nontarget language. In addition, this viewpoint suggests bilinguals have superior monitoring by constantly needing to be aware of linguistic context and adapting the target language [8,20]. In other words, being bilingual strengthens domain-general processes that, in turn, leads to advantages in cognitive control tasks, especially those that require processing of incongruent stimuli [21]. One task commonly used to index this ability is the dimension change card sort task (DCCS), where participants must first sort a set of cards by one dimension (e.g., color), and then re-sort by another (e.g., shape). The DCCS is labeled a measure of cognitive flexibility, and has been widely used in research on the bilingual advantage since it involves both inhibition and monitoring components [22].

Critics of the bilingual advantage have pointed out that there are major methodological issues in many studies including sampling as well as publication bias [7,10,18]. The most pervasive issue has been the conflation of bilingualism with other variables such as culture, minority or immigrant status, and socioeconomic status—indeed, this area has been likened to a “forest of confounding variables” [23].

With mounting contradictory evidence surrounding the bilingual advantage, it is pertinent to question what next steps are productive. Regardless of stance, most researchers acknowledge that bilingual advantages in cognitive control are unlikely to generalize to all bilinguals [1]. Therefore, instead of setting out to prove whether a bilingual advantage does or does not exist, research might instead characterize circumstances that do or do not lead to significant associations between bilingualism and cognitive control. The goal of such research is to develop testable hypotheses on certain aspects of bilingualism, cognitive control, or associated variables, with strong a priori theory concerning expected findings rather than post hoc explanations.

### 1.2. The Role of Attentional Fluctuations in Cognitive Control

Studies on the bilingual advantage in cognitive control frequently measure mean response time differences between monolingual and bilingual participants on tasks of cognitive flexibility, inhibitory control, and updating/monitoring. However, one aspect of cognitive control that, to our knowledge, has not been studied in this population is attentional fluctuations. Variability in response time on any task is presumed to represent attentional fluctuations, which have been shown to be an important cognitive trait that is predictive of cognitive performance in adults more generally [12,24,25]. This aspect of cognition has also been investigated in clinical populations (e.g., ADHD, dementia) who show heightened attentional fluctuations [26,27]. However, to date, research on attentional fluctuations in children—and especially bilingual children—is limited. One study on monolingual preschoolers showed that attentional fluctuations significantly predicted performance on that task (a go/no-go task), on a separate cognitive control task, and on laboratory measures of academic readiness [11]. Furthermore, cognitive flexibility mediated the association between attentional fluctuations and academic measures. The authors interpreted this to mean that consistency in attentional control may be a foundational aspect of EFs and cognitive control. In summary, consistency in attentional control has demonstrated associations with a wide range of cognitive performance, and it is likely an important underlying component of cognitive control.

To date, it is unclear whether bilingualism impacts attentional fluctuations. Previous meta-analyses synthesizing results on children and adults have measured several aspects of cognitive control including inhibition [2,4], cognitive flexibility [6], monitoring/working memory [3,6], and composite measures of EF [10]. Prior research has demonstrated associations between bilingualism and increased attentional control more generally (including both sustained and selective attention; [28]). Recently, however, selective attention has been shown not to be related to bilingual experience, with the authors claiming that “[t]he evidence that bilinguals are better than monolinguals at attentional control is equivocal at best” [29] (p. 1). However, to our knowledge, no study has investigated a potential bilingual advantage in attentional fluctuations specifically, as represented by variability in response time on cognitive control tasks. If one subscribes to the theory behind the bilingual advantage—that simultaneously managing two language representations enhances general attentional control—one would expect bilinguals to have lower attentional fluctuations. Given that attentional fluctuations have been found to contribute directly to measures of cognitive control [11], one might even hypothesize that bilingualism can relate to measures of cognitive control indirectly via attentional fluctuations. In other words, it is possible that attentional fluctuations could mediate or moderate between bilingualism and cognitive control. If one concurs with criticisms of the bilingual advantage, however, one would expect to see no such relationships if confounding variables (e.g., age, socioeconomic status) are adequately controlled for.

### 1.3. Defining and Measuring Bilingualism

Defining and measuring bilingualism is challenging, complex, and sometimes controversial. Researchers have used a number of metrics to determine bilingualism, including age of onset, context, proficiency, and identity. The age of onset of bilingualism, also called age of acquisition or exposure, generally distinguishes between “late” and “early” bilinguals, the latter of whom have been exposed to a second language in the first three years of life [30]. In behavioral studies, age of onset has been shown to contribute to later differences in second language vocabulary, grammar, and lexical access [31,32,33,34]. Neural differences in second language processing have also been observed when comparing early and late bilinguals [35,36,37]. However, it should be noted that these relationships are not always linear—some studies show that it is possible for late bilinguals to “catch up” to early bilinguals in terms of language performance [38]. 

In classifying bilinguals, studies have also placed an emphasis on the context in which language was acquired, distinguishing between formal and informal environments. These measurements take into account the use of second language in the home compared to school, with usage in a greater number of contexts generally believed to represent a greater degree of bilingualism [39]. Some researchers, however, have noted that second language acquisition rarely occurs strictly in institutional or naturalistic settings in isolation, and caution against drawing a definitive boundary between the two [39]. Researchers have also developed metrics to assess level of proficiency in both languages as a way of determining bilingualism. These methods typically assess receptive vocabulary in the individual’s first and second languages, and calculate proficiency of one language relative to the other [40]. Finally, some studies take into account the bilingual identity of the speaker by soliciting the self-reported ability to communicate in two languages [41]. For children, caregiver report of child language proficiency has been found to be concordant with laboratory language measures [42]. Of course, bilingual identity is often dependent upon the sociocultural context of use [39]. 

In sum, different methods across studies are often used in measuring bilingualism, which represents a replicability challenge for the field. Despite this, there are several points on which researchers converge. First, individuals are generally not dichotomously “monolingual” or “bilingual”—instead, bilingualism falls on a continuum [43]. Second, although historically examined separately, researchers have increasingly advocated for a conceptualization of bilingualism that is inclusive of second language learners [44]. 

### 1.4. Bilingualism and the Context of California

The evolving definition of bilingualism has also been influenced by globalization. Increased migration and access to more communication and information tools such as the Internet means the average individual has increasingly more contact with multiple languages [44]. As a consequence, the need for an inclusive and continuous definition of bilingualism is ever present. One geographic area where this need is especially salient is in California. California educates approximately one-third of the nation’s English language learners (ELLs), with 20.4% of children in the public school system meeting ELL status [45]. California has the largest percentage of immigrants of all U.S. States, with foreign-born residents representing more than 30% of the population in seven California counties [45]. In 2016, California responded to this growing population by passing Proposition 58, authorizing school districts to create dual-language immersion programs for both native and non-native English speakers. In other words, regardless of native language, students may learn in an English-only or a bilingual environment. California currently has 475 dual language schools, the highest of any U.S. state [46].

Children growing up in California may therefore be exposed to multiple languages in a number of environments: home, immersion schools, childcare, or community settings. Accordingly, measurement of bilingualism in these children must account for complex realities of second language exposure. Exact measurements of levels of proficiency are often difficult to obtain, given the lack of standardized assessments across the many languages spoken. One appropriate metric used in previous research is the amount of second language exposure, collapsed across different settings and evaluated by parent reports. Amounts of both home and school exposure to a second language is a strong predictor of children’s development of phonological processing, vocabulary, and grammar in that language [47,48]. Therefore, total years of second language exposure represents an ecologically valid and flexible measurement that is inclusive of second language learners immersed in a variety of settings. 

### 1.5. The Present Study

The present study seeks to extend the explosion of research on bilingualism and cognitive control to an aspect of cognition that has not been investigated: attentional fluctuations, as indexed by variability in response time. Additionally, we seek to add to previous mixed findings on the association between bilingualism and cognitive flexibility. We chose to measure cognitive flexibility given that it taps into both inhibition and monitoring, both of which are purported to be enhanced by language switching in bilinguals. Furthermore, cognitive flexibility has been found to mediate the association between attentional fluctuations and academic measures. We use amount of second language exposure as a measurement of bilingualism that is ecologically valid given the context of our participants, all of whom are in kindergarten in California public schools. The present study examined whether degree of bilingualism was associated with attentional fluctuations on a standardized task as well as with cognitive flexibility as measured by a different task. We specifically test two hypotheses presented in the literature about the relationship between bilingualism and EFs. If bilingualism indeed strengthens EFs, children that have more second language exposure should exhibit more cognitive flexibility and have lower attentional fluctuations. On the other hand, in line with research showing predominantly null results when confounding demographic factors are controlled for and standardized assessments of EF are used [7], one could expect no relationship between the degree of bilingualism and cognitive flexibility and attentional fluctuations. 

## 2. Methods

### 2.1. Participants and Procedure

**Figure 1:**
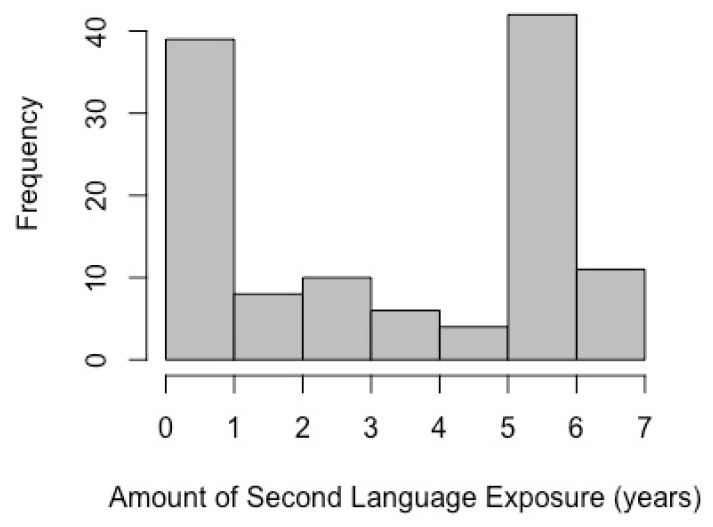
Histogram of the total amount of second language exposure in years for the overall sample (N = 120).

Participants were 120 children assessed in the fall of their kindergarten year. Families were recruited using flyers, email announcements, and community events from Northern California public schools as part of a larger longitudinal study investigating language and literacy acquisition. The present analyses used cross-sectional data from the first timepoint. Children were screened for diagnoses of any neurological disorders, and they had normal or corrected-to-normal vision. Children were assessed on all measures directly by trained assessors, and questionnaires were administered to parents to obtain demographic data. All study procedures were reviewed and approved by the university institutional review board (UCSF IRB #13-11958), and all participating families provided informed consent. Families were compensated $40 for participating in the research study. English was the native language for all children in the present analysis, and all were born in the United States. Of the overall sample, 104 had been exposed to at least one other language than English, and 16 had no second language exposure (see Figure 1).

### 2.2. Measures

#### 2.2.1. Demographic Characteristics.

Age, gender, ethnicity, race, and immigrant status of the participating children were collected through parent questionnaires (see Table 1). Socio-economic status (SES) was indexed as the highest level of education in years each parent had attained. Education levels of both parents were averaged to create one SES metric.

#### 2.2.2. Second Language (L2) Exposure and Age of Acquisition (AoA)

Second language (L2) exposure and age of acquisition (AoA) were determined from language background questionnaires given to parents. Parents were specifically prompted to provide information on language exposure resulting from babysitting/daycare experience, immersion preschool or kindergarten, or home exposure from parents or family members. If parents indicated the child had experienced some exposure to any language other than English, information on the age of first exposure was collected as well as the exposure length. Information was also collected on which language the child was exposed to. Consecutive experiences with different languages throughout the child’s life were added up. L2 exposure and AoA were then calculated in terms of years, see Table 1.

#### 2.2.3. Cognitive Flexibility

Cognitive flexibility, the ability to shift attention between dimensions or tasks, was indexed by the NIH Toolbox Dimensional Change Card Sort Test (DCCS) administered on an iPad. During the task, participants were presented with two target pictures and were asked to select the picture that was congruent with the dimension rule (shape or color). Participants needed to navigate and shift between the two-dimension rules across trials. NIH Toolbox DCCS has shown high test–retest reliability (Intraclass Correlation Coefficients = 0.86–0.95, [49]). Age-corrected standard scores were used in analysis.

#### 2.2.4. Attentional Fluctuations.

Attentional fluctuations were indexed using the tasks of executive control (TEC), a computerized task that combined *n*-back and go/no-go paradigms to tap into working memory, inhibitory control, and sustained attention—elements of cognitive control [50]. Our sample completed four sequential tasks combining levels of working memory load (0- or 1-back) and inhibitory control (no inhibit or inhibit). Intraindividual coefficient of variation (ICV) was output from the task as a score and was used in analysis, as it had been used as a reliable measure of attentional fluctuations in previous studies [12]. ICV was calculated for each individual as their standard deviation of response time divided by that individual’s mean response time. This score was standardized into an age-corrected T-score, and elevated T scores indicated greater than expected variability in response time.

### 2.3. Analytic Plan

All analyses were conducted and plots created in R version 3.3.2. Descriptive statistics (mean, standard deviation, and percentages) were computed for study variables for all participants as well as separately for those with some amount of second language exposure (N = 104) and those with no second language exposure (N = 16; see Table 1). All study variables fell within values of skewness and kurtosis that indicated no extreme violations of normality (between −4 and +4). Bivariate correlations were calculated for continuous variables, and t-tests were calculated for categorical variables (gender, race, and ethnicity). For t-tests and regression, race was re-coded into a dummy variable (1 if White, 0 otherwise). Regression analyses were used to determine whether there were significant associations between L2 exposure and attentional fluctuations, as well as between L2 exposure and cognitive flexibility, controlling for AoA, race, and SES. Both correlation and regression analyses only included participants with some amount of L2 exposure (N = 104). Additionally, t-tests were conducted to compare participants with no L2 exposure (N = 16) and participants with consistent L2 exposure from birth to present (N = 24). Although these sample sizes were small, we performed this analysis to see if differences on attentional fluctuations or cognitive flexibility would emerge when only including participants at each extreme of the bilingualism continuum. According to a power analysis conducted in G*Power 3.1 [51], this analysis had power to detect large (power = 0.79) effects but limited power to detect medium (0.45) and small (0.15) effects using an alpha of 0.05 and effect sizes of 0.2, 0.5, and 0.8 to represent small, medium, and large effects, respectively. Finally, using BayesFactor R package [52], Bayes factor analyses were conducted in order to establish the strength of evidence for the present hypotheses and to establish whether any potential non-significant results of L2 exposure originated in true null-effects or in insensitivity of our data [53]. Bayes factor indicated how many times better (or worse) a particular model accounted for the data than the null model. 

## 3. Results

### 3.1. Descriptive Statistics and Bivariate Correlations

**Figure 2:**
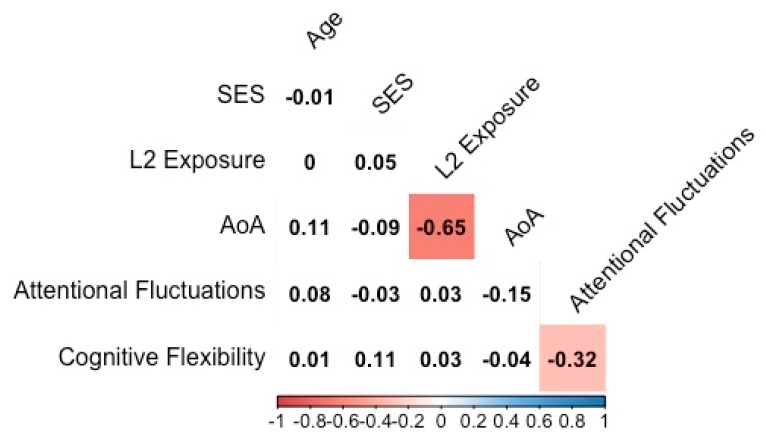
Bivariate correlations between age, SES, years of second language (L2) exposure, age of acquisition (AoA), attentional fluctuations, and cognitive flexibility. Correlation coefficients are displayed, with directionality and strength of each relationship coded in the bottom color bar. All significant correlations are colored.

Descriptive statistics of study variables are displayed in Table 1, and correlations among study variables are shown in Figure 2. There were no significant correlations between L2 exposure and attentional fluctuations or between L2 exposure and cognitive flexibility (both *p*s > 0.05). Age of acquisition also did not significantly correlate with either attentional fluctuations or cognitive flexibility (both *p*s > 0.05). There was a significant, negative correlation between attentional fluctuations and cognitive flexibility (*p* = 0.001). There were no significant differences in study variables based on gender (all *p*s > 0.05). There were significant group differences according to race, where nonwhite participants had significantly higher L2 exposure (*t*_(102)_ = 3.01, *p* = 0.003) and younger age of acquisition (*t*_(102)_ = −2.12, *p* = 0.037). Age did not significantly correlate with L2 exposure.

### 3.2. Regression Analyses with Bilingualism as a Continuous Variable

Overall, the model predicting attentional fluctuations was not significant (Table 2; R^2^ = 0.023, F(4,88) = 0.52, *p* = 0.72). L2 exposure did not significantly predict attentional fluctuations according to regression analyses (β = −0.66, *t*_(99)_ = −0.95, *p* = 0.34), controlling for covariates (AoA, race, and SES). A separate model predicting cognitive flexibility was also not significant overall (Table 2; R^2^ = 0.015, F(4,88) = 0.33, *p* = 0.86). In this model, L2 exposure did not significantly predict cognitive flexibility (β = 0.862, t_(99)_ = −0.458, *p* = 0.649), controlling for covariates. Age of acquisition, race, and SES did not significantly predict either attentional fluctuations or cognitive flexibility T-scores.

Both models were replicated with Bayes factor analyses, testing all model combinations and their predictive success relative to an intercept-only (null) model with default priors. For both attentional fluctuations and cognitive flexibility, L2 exposure, as our variable of interest, proved to predict the data substantially [54] *worse* than an intercept-only model (*B* = 0.22 in both cases).

### 3.3. Group Comparison with Bilingualism as a Categorical Variable

**Figure 3:**
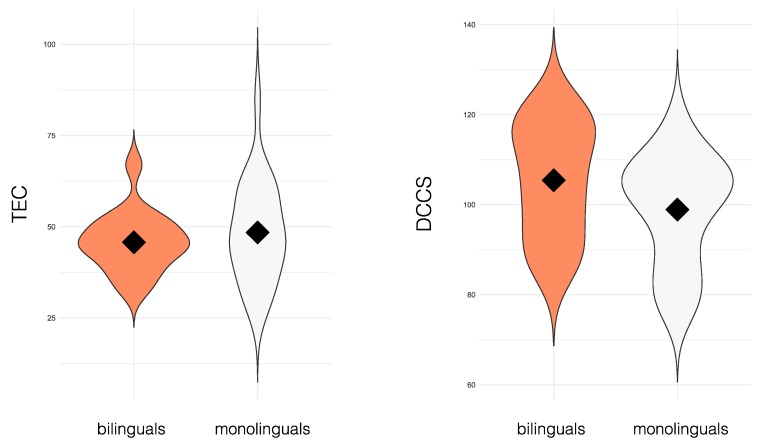
Attentional fluctuations (tasks of executive control (TEC), left panel), and cognitive flexibility (dimension change card sort task (DCCS), right panel) for bilingual (*N* = 24) and monolingual (*N* = 16) participants. Black diamonds represent group means, and the violin plot outlines illustrate the density of the data, i.e., the width of the shaded area represents the proportion of the data located there.

There were no significant group differences in attentional fluctuations between children who had no L2 exposure, i.e., monolinguals (*N* = 16), and those who had been continuously exposed to a second language since birth, i.e., simultaneous bilinguals (*N* = 24; *t*_(38)_ = −0.58, *p* = 0.56, Figure 3). There were also no significant differences in cognitive flexibility between these two groups (*t*_(38)_ = 1.6403, *p* = 0.11, Figure 3). Bayes factors were computed for both comparisons, resulting in *B* = 0.38 for attentional fluctuations, and *B* = 0.89 for cognitive flexibility. In both cases, these analyses provided only anecdotal evidence [54] for a true null effect, the strength of the evidence plausibly stemming from the small sample size included. 

## 4. Discussion

The present study sought to replicate prior findings on a bilingual advantage in cognitive flexibility as well as investigate the association of bilingualism with attentional fluctuations. Previous studies have not considered consistency in attentional control when exploring the bilingual advantage. Our results showed no evidence for a bilingual advantage in either cognitive flexibility or attentional fluctuations, with bilingualism investigated both continuously and categorically. Furthermore, the nonsignificant results obtained with classical frequentist statistical methods were confirmed to stem from true null effects of bilingual experience on attentional fluctuations and cognitive flexibility by means of Bayes factor analyses.

Correlational analyses did not show any significant associations between continuous variables indexing bilingualism (L2 exposure and age of acquisition) and both outcome variables (cognitive flexibility and attentional fluctuations). Amount of L2 exposure also did not correlate with SES—this was likely because of the restricted range of our sample consisting of only middle- to high-SES families. Regression analyses also showed that L2 exposure did not significantly predict attentional fluctuations or cognitive flexibility, controlling for age of acquisition, race, and SES. Using Bayes factor analyses, we showed that L2 exposure predicted the data substantially worse [54] than an intercept-only model, adding to the evidence of no relationship between bilingualism and attentional fluctuations or cognitive flexibility. We included race and SES as covariates, given that they often confounded reported bilingual advantages [55,56]. In the present study, race and SES were not significantly associated with cognitive flexibility or attentional fluctuations—however, race was associated with L2 exposure and AoA. We interpret the lack of associations with race, SES, and study outcomes to be due to our specific sample, and we still encourage future studies to treat these variables as confounds.

The hypothetical link between bilingualism and cognitive flexibility derives from the notion that bilinguals must constantly attend to one language and flexibly shift to the other language when prompted. This practice in shifting the relative activation of language systems in working memory is believed to lead to improved performance on cognitive flexibility tasks [57,58]. However, the present study found no evidence for a bilingual advantage in cognitive flexibility, concurring with previous null findings on this association [4,7,18,21,55,59,60,61]. Our results may not match those studies purporting a bilingual advantage because of sample size—Paap et al. [7] found that a bilingual advantage in cognitive flexibility was more likely in studies with smaller samples (*N* < 30). However, it is notable that our categorical analysis included a similarly small sample size and still found no evidence for a bilingual advantage. Our null results could also be attributable to measuring bilingualism continuously—indeed, previous studies measuring degree of bilingualism failed to replicate findings from studies that indexed bilingualism dichotomously [29,61,62]. To address this, we split our sample into a monolingual and bilingual group, with the bilingual group defined as those with continuous second language exposure since birth. This analysis still failed to find a group difference on cognitive flexibility. There may be other influential sample characteristics if a bilingual advantage is more likely to occur, such as age. Previous studies have suggested that a bilingual advantage on cognitive flexibility is more likely to present in children and the elderly, as opposed to young adults [63]; however, we still did not find this result in our sample of children, which is in line with other studies showing null results on a bilingual advantage in children [55,64].

Our study also did not find any associations between bilingualism and attentional fluctuations. Our interest in investigating this association arose out of the theory that the bilingual experience enhances overall attention processes (including selective and sustained attention) in addition to cognitive flexibility [22,65]. According to this theory, bilinguals must constantly attend to two language systems and direct attention to task-relevant information to select which language to deploy [22,65]. If this theory is true, one would expect the enhancement of attention processes to include reduced lapses in sustained attention (i.e., lower attentional fluctuations). Furthermore, given that attentional fluctuations are shown to underlie accuracy on cognitive tasks [12], it may be that attentional fluctuations could modulate or mediate the relationship between bilingualism and cognitive flexibility. Indeed, attentional fluctuations and cognitive flexibility were significantly correlated in our sample, with more fluctuations in attention on one task associated with lower performance on the cognitive flexibility task. However, given that bilingualism did not relate to either of these outcome variables, we did not investigate any mediating or moderating relationships.

The present findings—as well as previous studies on this topic—are limited in interpretation because there is a lack of straightforward accounts on mechanisms underlying the supposed bilingual advantage. There is a general account that management of two languages enhances cognitive control and attention in bilinguals, but there is a lack of specific and falsifiable hypotheses in this area [66]. Indeed, one researcher has likened studies on the bilingual advantage to “a hunt for treasure by randomly digging holes in uninhabited islands” [67] (p. 337). An analogous debate is that on mechanisms underlying transfer in cognitive training programs [68], it is unclear how transfer occurs, limiting research findings to post hoc explanations. We acknowledge that the present study also operates on a general theory connecting bilingualism to cognitive flexibility and attention. We do not investigate any specific mechanistic account, except with intention to explore whether attentional fluctuations might mediate or moderate between bilingualism and cognitive flexibility. Future studies—and the field as a whole—would benefit from testing specific accounts of language control and cognitive transfer [67].

Several other limitations of the present study should be acknowledged. First, we used summary scores (amount of L2 exposure, age of acquisition) for our measurement of bilingualism. This did not allow for more fine-grained analysis of the frequency of language switching in individual children, which may be important given theory linking this variable to cognitive advantages. One promising new way to index bilingual language switching behavior involves collecting ecological momentary assessment data on this variable several times a day with smartphones, a methodology that has been recently developed [69].

Another limitation pertaining to measurement was that we were unable to obtain standardized proficiency measures for the range of second languages in our sample. In our categorical analysis, we classified bilinguals as those with second language exposure since birth; however, we acknowledge that this may not necessarily map onto language proficiency. In order to address this issue, we performed an additional set of analyses (see Supplementary Materials) where only Spanish L2 participants were considered for whom L1 and L2 proficiency scores (Peabody Picture Vocabulary Test, PPVT [70]; Test de Vocabulario en Imagenes Peabody, TVIP [71]) were available (*N* = 37). We found no significant correlations between children’s L2 proficiency, attentional fluctuations, or cognitive flexibility. How balanced participants were in English and Spanish (determined by means of language dominance index, see Supplementary Materials for further information) also did not correlate with their TEC or DCCS scores. 

A second limitation is that our sample may restrict the generalizability of our findings. Our participants consisted of children who were all native English speakers, were born in the U.S., resided in Northern California, and were moderate-to-high SES. Results might be different for children of low SES, immigrant status, or in different regions of the country or world.

In summary, this study adds to null findings on the bilingual advantage in cognitive flexibility. Our results also found no evidence for an association between bilingualism and attentional fluctuations—an important contributor to cognitive performance. Because of this, we did not investigate whether attentional fluctuations moderated or mediated between bilingualism and cognitive flexibility. We found the same results when investigating bilingualism continuously and categorically. We interpret our results to mean that a bilingual advantage is not evident for individuals with characteristics and circumstances similar to our sample. Despite the present null results, we want to emphasize that ours, along with any other results reporting null effects of the bilingual experience on general cognition, should in no way discourage the development of dual-language proficiency and second language learning. Knowing another language brings countless advantages outside of the realms of cognition, which include (but are not limited to) one own’s cultural expansion, broadening of the horizons, open-mindedness and expanded communicative abilities. Future studies should test more specific mechanistic accounts of the interplay between language and cognition, as well as the value of language outside of cognitive domains, which will be informative for understanding the world’s growing population of bilingual individuals.

## Figures and Tables

**Table 1:** Descriptive statistics for covariates and variables of interest in the overall sample and by language exposure group. Specifically, we compare individuals with some level of L2 exposure (N = 104), and of these, individuals who have been exposed to L2 since birth (N = 24), compared to individuals with no L2 exposure (N = 16) and the overall pooled sample (N = 120).

	Overall Sample (N = 120)	No L2 Exposure (N = 16)	Some L2 Exposure (N = 104)	L2 Exposure Since Birth (Subsample) (N = 24)
	Mean (SD)/%	Mean (SD)/%	Mean (SD)/%	Mean (SD)/%
**Age (y)**	5.68 (0.36)	5.83 (0.36)	5.66 (0.36)	5.63 (0.37)
**Parent Education (y)**	16.6 (2.00)	16.1 (1.95)	16.7 (2.01)	16.5 (2.39)
**Attentional Fluctuations**	47.1 (10.8)	45.8 (8.3)	47.3 (11.2)	48.0 (13.5)
**Cognitive Flexibility**	101 (13.3)	105 (12.5)	101 (13.4)	99 (12.1)
**Gender (% male)**	55.4	56.2	54.8	58.3
**Ethnicity (%)**				
Hispanic/Latino	17.5	6.25	19.2	16.7
Not Hispanic/Latino	82.5	93.8	80.8	83.3
**Race (%)**				
Asian	22.5	12.5	24.0	50.0
Black	1.67	0.00	1.92	0.00
White	49.2	56.3	48.1	20.8
Multiracial	22.5	25.0	22.1	20.8
Unknown	4.17	6.25	3.85	8.33
**L2 Exposure (y)**			3.19 (2.40)	5.63 (0.37)
**Age of Acquisition (y)**			0.83 (1.49)	0.00 (0.00)
**Second Language (% of sample)**				
Spanish			53.3	29.2
Cantonese			22.2	25.0
Mandarin			7.78	4.17
Arabic			3.33	0
French			2.22	0
Ilocano			2.22	4.17
Other			8.89	37.5

**Table 2:** Regression models for attentional fluctuations (Model 1) and cognitive flexibility (Model 2).

	**Model 1 (Predicting Attentional Fluctuations)**			
	*Estimate*	*Std. Error*	*t-value*	*p-value*
**(Intercept)**	54.14	10.31	5.25	1.04 × 10 ^−6^ ***
**L2 Exposure**	−0.66	0.69	−0.95	0.34
**AoA**	−1.15	0.90	−1.28	0.20
**Race**	−1.14	2.32	−0.49	0.63
**SES**	−0.15	0.58	−0.25	0.80
	**Model 2 (Predicting Cognitive Flexibility)**			
	*Estimate*	*Std. Error*	*t-value*	*p-value*
**(Intercept)**	88.17	13.34	6.61	3.00 × 10 ^−9^ ***
**L2 Exposure**	0.044	0.89	0.05	0.96
**AoA**	−0.081	1.20	−0.07	0.95
**Race**	1.48	3.02	0.49	0.63
**SES**	0.70	0.76	0.91	0.36

* *p* < 0.05, ** *p* < 0.01, and *** *p* < 0.001.

## Supplementary Materials

The following are available online at https://www.mdpi.com/2076-328X/9/5/58/s1.
