# Supplementary material for: Attentional Fluctuations, Cognitive Flexibility, and Bilingualism in Kindergarteners
Source: Behav Sci (Basel). 2019 May 24;9(5):58. doi: 10.3390/bs9050058 (PMC6562579; doi:10.3390/bs9050058)
Supplement: Supplementary file 1 [file behavsci-09-00058-s001.pdf]

Supplementary Analyses

Language Dominance Index. Language dominance index was calculated in order to determine how proficient the L2 Spanish speakers were in their L2 along with how balanced in English and Spanish they were. We used standard scores on the Peabody Picture Vocabulary Test, 4th Edition (PPVT-4) [70] as an index of English language proficiency, and standard scores on the Test de Vocabulario en Imagenes Peabody (TVIP) [71] as an index of Spanish language proficiency. In these tests, considered a measure of receptive vocabulary knowledge, participants were asked to pick one out of four pictures that best described a word stated by the examiner for each question. Language dominance was then calculated using the following formula:

$$language\ dominance\ index\ = \frac{L1-L2}{L1+L2}.$$

Table S1. Correlations between Spanish language proficiency (as indicated by a language dominance index and Spanish receptive vocabulary raw scores), years of second language exposure, age of acquisition, attentional fluctuations, and cognitive flexibility. Higher dominance index is indicative of greater English proficiency relative to Spanish.

	1	2	3	4	5
**1.** Language Dominance Index	-				
**2.** Spanish Receptive Vocabulary (raw)	−0.91 ***	-			
**3.** Years of Second Language Exposure	−0.38 *	0.45 **	-		
**4.** Age of Acquisition of Second Language	0.20	−0.19	−0.57 ***	-	
**5.** Attentional Fluctuations (T Score)	−0.19	0.21	0.05	−0.09	-
**6.** Cognitive Flexibility (Standard Score)	0.19	−0.11	−0.04	−0.01	−0.32 ***

$^{+}$ = $p < 0.10$; * = $p < 0.05$; ** = $p < 0.01$; *** = $p < 0.005$.
